# Supplementary material for: Impact of workplace bullying on work engagement among early career employees
Source: PLoS One. 2023 Oct 30;18(10):e0285345. doi: 10.1371/journal.pone.0285345 (PMC10615286; doi:10.1371/journal.pone.0285345)
Supplement: Attachment — Submitted filename: reviewer comment.docx [file pone.0285345.s001.docx]

**Comment 1:**

The revised manuscript looks promising, but it still requires more streamlining on the grounds that can warrant its publication. The authors suggest that it is interesting to find out how bullied employees are going to behave when it comes to knowledge sharing especially when already plenty of research on negative effects of bullying is available. What is missing in the manuscript is why the specific effects authors have investigated are interesting and relevant in view of a host of existing literature on bullying effects. More justification in terms of the characteristics of the sample (sector, tenure, and country) that makes it a unique and meaningful contribution should be added for further improvement.

**Response:**
We are grateful to receive such comprehensive feedback. Thank you for sparing time to review the article. We have incorporated all the points keeping in in view all the details and tried to improve it in all the ways.

**Comment 2:**

In my view, it is a key requirement for the authors to identify and describe a theoretical contribution that warrants publication. So far, the authors pointed out that “It is interesting to find out how bullied employees are going to behave when it comes to knowledge sharing within the organization” (81-83), while conceding that there is already plenty of research on negative effects of bullying. The authors largely miss to elaborate, why the specific effects they investigated are interesting and relevant in view of a host of existing literature on bullying effects. Moreover, I am not convinced that the characteristics of the sample (sector, tenure, and country) constitute a meaningful contribution, as the authors do not explain why (and test if) these characteristics might influence the investigated effects.

**Response:**

Thank you for the in-depth feedback. The comments have been incorporated in the introduction section.

**Comment 3:**

First, the authors could elaborate more on their DVs and why they are novel in bullying research and/or contribute to solve existing theoretical or practical puzzles. For instance, the authors could consider framing Employee Engagement as a potential “self-protective” behavior buffering against (future) bullying, whereas Employee Silence allows bullies to get away with it and thus might even exacerbate abusive behavior in a vicious cycle.

**Response:**

The comments were very enlightening and added a lot to the author’s knowledge. The response to the comment is now added in the discussion section.

**Comment 4:**

Second, the authors could elaborate more on the moderator and how it contributes to explain and reconcile empirical inconsistencies in the field.

**Response:**

The explanation of the moderating variable i.e., psychological breach and how it contributes to explain and reconcile empirical inconsistencies in the field has been added in the introduction section.

**Comment 5:**

Third, the authors could elaborate on the context (Pakistani banking sector), going beyond claiming that this context has never been looked at, but arguing why comparability with previously studied contexts is severely limited and, why the context of the present study is relevant to expanding our overall knowledge in this field.

**Response:**

We have added the required information about the Pakistani banking industry especially in the introduction section where the model and variables were explained.

**Comment 6:**

The authors describe both the independent and the dependent variables in a comprehensive manner. Moreover, drawing on relevant and established theory (COR), the authors introduce a compelling psychological mechanism explaining their findings.

**Response:**
Thank you for the positive comment.

**Comment 7:**

However, I would like to point to logical flaws in the theoretical model.
First, I have some doubts about described causal links between mediators (i.e., employee silence, knowledge sharing) and the DV (i.e., work engagement). While all three variables are certainly highly correlated, I am not convinced about the described direction of the effect (i.e., that behaviors like silence and knowledge sharing lead to a more perception-based variable like work engagement). Second, I am not convinced of the interaction effect of Breaching of a Psychological Contract and workplace bullying on employee engagement, given that the authors miss to explain the hypothesized interaction between PCB and bullying, including the simple effects of bullying if PCB is high vs. low.
To address these concerns, I encourage the authors to elaborate more on the causal links and the interaction effect they seek to establish.

**Response:**
Thank you for the very productive feedback. The above-mentioned has been incorporated at multiple places throughout the document especially where the hypotheses were developed.

**Comment 8:**

The sample is described with due thoroughness.

As conceded by the authors, a major limitation is the design of the present study. Therefore, the authors cannot draw conclusions about the causality of the relationships described in the theoretical model, limiting the possibility of making a true contribution.
In my view, the present manuscript could benefit greatly from adding another study. For instance, a study with multiple points of measurement (e.g., event sampling) would help to gain insight into the direction of the effects (e.g., one could hypothesize that bullying leads to Employee Silence which in turn exacerbates bullying). Moreover, the authors may overcome the common-source bias of the present study in another study.

**Response:**

Thank you for enlightening us and showing us future direction. The above comments will be incorporated when a new research will be conducted; building on to this research in the future.
